# Waterlogging Stress Induces Antioxidant Defense Responses, Aerenchyma Formation and Alters Metabolisms of Banana Plants

**DOI:** 10.3390/plants11152052

**Published:** 2022-08-05

**Authors:** Ee Yang Teoh, Chee How Teo, Nadiya Akmal Baharum, Teen-Lee Pua, Boon Chin Tan

**Affiliations:** 1 Centre for Research in Biotechnology for Agriculture (CEBAR), Universiti Malaya, Kuala Lumpur 50603, Malaysia; 2 Department of Cell and Molecular Biology, Faculty of Biotechnology and Biomolecular Sciences, Universiti Putra Malaysia (UPM), Serdang 43400, Malaysia

**Keywords:** abiotic stress, banana, crop improvement, waterlogging, transcriptomics

## Abstract

Flooding caused or exacerbated by climate change has threatened plant growth and food production worldwide. The lack of knowledge on how crops respond and adapt to flooding stress imposes a major barrier to enhancing their productivity. Hence, understanding the flooding-responsive mechanisms of crops is indispensable for developing new flooding-tolerant varieties. Here, we examined the banana (*Musa acuminata* cv. Berangan) responses to soil waterlogging for 1, 3, 5, 7, 14, and 24 days. After waterlogging stress, banana root samples were analyzed for their molecular and biochemical changes. We found that waterlogging treatment induced the formation of adventitious roots and aerenchyma with conspicuous gas spaces. In addition, the antioxidant activities, hydrogen peroxide, and malondialdehyde contents of the waterlogged bananas increased in response to waterlogging stress. To assess the initial response of bananas toward waterlogging stress, we analyzed the transcriptome changes of banana roots. A total of 3508 unigenes were differentially expressed under 1-day waterlogging conditions. These unigenes comprise abiotic stress-related transcription factors, such as ethylene response factors, basic helix-loop-helix, myeloblastosis, plant signal transduction, and carbohydrate metabolisms. The findings of the study provide insight into the complex molecular events of bananas in response to waterlogging stress, which could later help develop waterlogging resilient crops for the future climate.

## 1. Introduction

Flooding is one of the most significant threats to food production and economic growth worldwide. It affects 17 million km^2^ of land surface annually [1], causing severe damage to agricultural crop production. The frequency of flooding events is expected to increase in the near future, especially in Southern Asia countries [2], due to increasingly erratic rainfall patterns exacerbated by climate change [3]. For example, in Malaysia, the flooding event in December 2006 amounted to 18.9 million USD of agriculture loss and damage, affecting the arable lands and farmers [4]. Similarly, the flooding event in the Mississippi River and Midwest in 2019 resulted in a cumulative loss of USD 6.9 billion, mainly affecting agriculture [5]. These occurrences encourage scientific initiatives to develop flood-tolerant varieties to mitigate agricultural losses and improve global crop production.

Waterlogging is part of the flooding stress, where the soil is oversaturated with water, and only plant roots are surrounded by water. Oxygen partial pressure and gas diffusion in waterlogged soil gradually decline, disrupting plant root function and normal cellular processes and metabolisms [6]. Consequently, the nutrient uptake by plants dramatically decreases, leading to plant starvation [7]. Several studies have shown that waterlogging is a significant yield-limiting factor for crop yield and growth [8,9]. For instance, waterlogging reduced the yield of corn by about 4.7% each day for up to seven days compared to well-watered corn [10]. Therefore, it is crucial to understand the plant molecular mechanisms in response to flooding stress to mitigate its effects on plants.

Plants have developed multiple and interrelated signaling pathways to modulate stress-responsive genes, leading to morphological, physiological, and metabolic changes [11]. At the morphological level, plant growth, biomass, and yield decreased under waterlogged conditions [12]. In addition, the root architecture of plant species is usually altered in response to waterlogging stress. For instance, adventitious roots and aerenchyma formation were observed in waterlogged maize plants [13]. The changes in the root system could help mitigate oxidative stress and promote oxygen movement to roots under hypoxic stress [14]. At the physiological and biochemical levels, waterlogging stress induces stomatal closure, osmolyte accumulation, and reactive oxygen species (ROS) production while reducing the plant’s photosynthetic capacity [15,16]. In the presence of ethylene, waterlogging stress enhances respiratory burst oxidase homolog (RBOH) expression, leading to the increase of ROS and hydrogen peroxide (H_2_O_2_) levels [17]. Consequently, plants activate their antioxidant defense machinery to scavenge excessive ROS. At the molecular level, genes and proteins involved in hormonal signal pathways and carbohydrate and energy metabolisms have been reported to play significant roles in waterlogging stress responses through the N-end rule pathway [18]. These findings show that plants adopt different strategies to defend themselves from waterlogging stress.

Recently, much progress has been made to decipher the plant-responsive mechanisms against flooding stress [17,19]. However, although the previous findings contribute to our understanding of the adverse effects of flooding stress on plants, how crops respond and adapt to it remains largely unknown. Hence, understanding the flooding-responsive mechanisms of crops is indispensable for developing new flooding-tolerance varieties.

The banana is a commercially important cash crop as its nutritional value is higher than other tropical fruits [20]. It ranked among the top ten crops in terms of yield and calories produced, with about 119 million tons produced globally in 2020 [21]. In Malaysia, the banana cultivar Berangan is one of the most popular cultivars and has the highest commercial value in the Southeast region [22]. However, global extreme precipitation events are a major constraint for banana yield and negatively affect its productivity [23]. Furthermore, the predicted rise in the earth’s global temperature is likely to aggravate flooding effects [24,25], fostering a global decline in banana yield. Therefore, improving banana production and the quality of this economically valuable fruit is vital.

We used bananas (*Musa acuminata* cv. Berangan) as a model to elucidate waterlogging stress responses. The main objective of this study was to understand how waterlogging stress causes changes at the morpho-biochemical and molecular levels to explore the early stress-responsive mechanisms in bananas. We determined the morphological and biochemical changes of banana plants at different time points of waterlogging treatment. In addition, several genes involved in waterlogging signaling and regulation in the early stage of waterlogging were also identified using transcriptomics and bioinformatics approaches. The novelty of our study is that genes related to oxygen sensing and hormone signaling are altered at the early stage of waterlogging stress. Such findings are critical and will serve as a foundation for developing stress-tolerant bananas.

## 2. Results

### 2.1. Waterlogging Stress Influences Banana Growth

**Figure 1:**
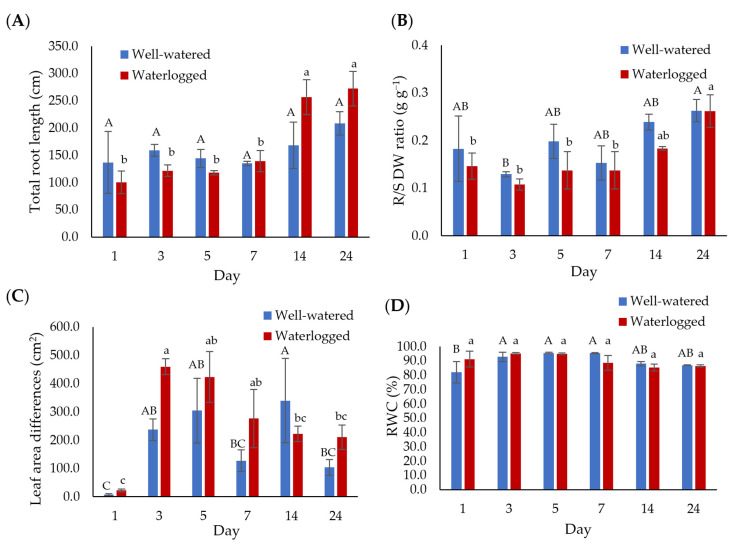
The growth of well-watered and waterlogged banana plants was measured in terms of (**A**) total root length, (**B**) root-to-shoot (R/S) dry weight (DW) ratio, and (**C**) leaf area differences. (**D**) The effect of waterlogging on relative water content (RWC) was assessed between well-watered and waterlogged leaf samples. Data are presented as means ± standard error from nine independent biological replicates. Different capital letters indicate a significant difference between time points within well-watered samples, while different lowercase letters indicate a significant difference between time points within waterlogged samples (*p* < 0.05).

The growth of well-watered and waterlogged banana plants was measured at 1, 3, 5, 7, 14, and 24 days (Figure 1). Waterlogging stress impacted the banana root length, producing the highest root length of 272.4 cm after 24 days of waterlogging (Figure 1A). The root-to-shoot (R/S) dry weight (DW) for well-watered samples increased from 0.18 g g^−1^ on day 1 to 0.26 g g^−1^ on day 24 (Figure 1B; Table S1). In contrast, a significant increase in the R/S DW ratio for waterlogged samples was observed between days 7 (0.15 g g^−1^) and 24 (0.26 g g^−1^) (Figure 1B). The waterlogged plants generally had a larger leaf area than well-watered plants. The leaf area differences were highest on day 14 for well-watered plants (339.90 cm^2^) and on day 3 for waterlogged bananas (459.3 cm^2^) (Figure 1C). However, the differences between well-watered and waterlogged samples were not significant. Although we did not notice any visible damage to plants, the leaves of the waterlogged banana showed yellowing after 24 days of waterlogging (Figure S1). The relative water content (RWC) for both treatments was similar throughout the experiments, ranging between 82% and 95% (Figure 1D).

### 2.2. Waterlogging Induces Adventitious Roots and Aerenchyma Formation

**Figure 2:**
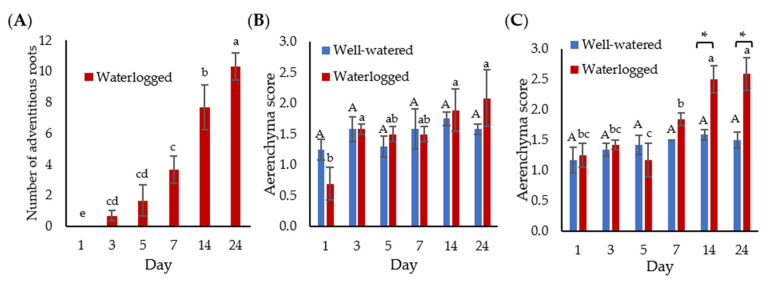
Adventitious root and aerenchyma formation. (**A**) The number of adventitious roots in well-watered and waterlogged banana plants. The mean aerenchyma score of (**B**) 5 cm from the root tip and (**C**) 5 cm from the root base. The results represent the mean ± standard error of the mean of nine independent biological replicates with three technical replicates each. Different capital letters indicate a significant difference between time points within well-watered samples. Different lowercase letters indicate a significant difference between time points within waterlogged samples. Asterisk (*) indicates a significant difference between well-watered and waterlogged samples at the same time point (*p* < 0.05).

The waterlogged banana plants started to produce adventitious roots after three days of waterlogging, with the highest number of adventitious roots (10 roots) recorded after 24 days of waterlogging (Figure 2A). In contrast, the well-watered plants did not grow adventitious roots throughout the experiment.

The well-watered bananas recorded an average aerenchyma score of 1.17 to 1.75 throughout the experiment (Figure 2B,C). The aerenchyma size of the root tip did not show a significant difference between well-watered and waterlogged samples (Figure 2B). However, the aerenchyma score of the root base of waterlogged samples was significantly higher than well-watered samples on days 14 and 24 (Figure 2C).

### 2.3. Malondialdehyde, Proline and Hydrogen Peroxide Contents Changed under Waterlogging Stress

**Figure 3:**
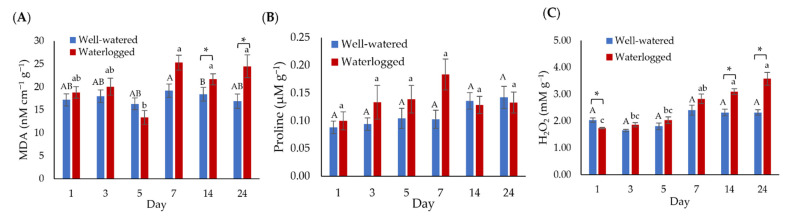
Proline and lipid peroxidation changes in the well-watered and waterlogged banana plants. (**A**) Malondialdehyde (MDA), (**B**) proline contents, and (**C**) hydrogen peroxide (H_2_O_2_) of banana roots. Error bars indicate the standard error of nine independent replications with three technical replicates each. Different capital letters indicate a significant difference between time points within well-watered samples. Different lowercase letters indicate a significant difference between time points within waterlogged samples. Asterisks (*) indicate a significant difference between well-watered and waterlogged samples at the same time point (*p* < 0.05).

Malondialdehyde (MDA) and proline contents were determined on banana root samples (Figure 3A,B). MDA and proline contents remained relatively like well-watered samples throughout the first five days of the waterlogging treatment. However, significant changes in MDA content were observed after 14 days of waterlogging treatment. Similarly, the H_2_O_2_ content of waterlogged samples was significantly higher than well-watered samples on day 14 and day 24 of waterlogging (Figure 3C).

### 2.4. Waterlogging Stress Enhances Antioxidant Defense Systems

**Figure 4:**
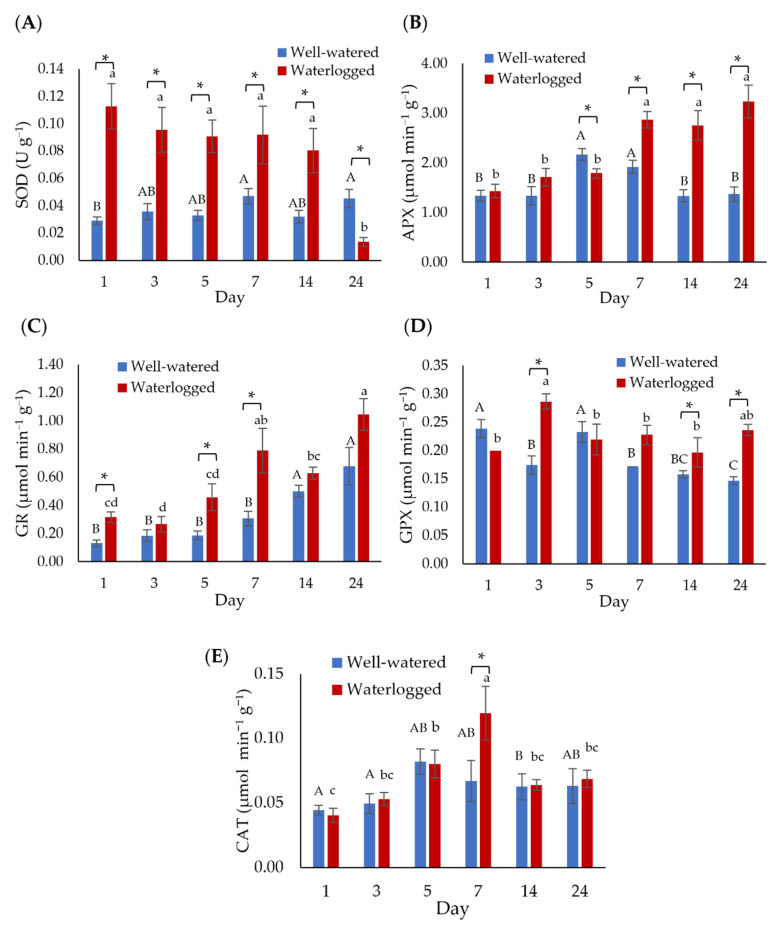
Activities of (**A**) superoxide dismutase (SOD), (**B**) ascorbate peroxidase (APX), (**C**) glutathione reductase (GR), (**D**) glutathione peroxidase (GPX), and (**E**) catalase (CAT) in banana plants in response to waterlogging. Error bars indicate the standard error of nine independent replications with three technical replicates each. Different capital letters indicate a significant difference between time points within well-watered samples. Different lowercase letters indicate a significant difference between time points within waterlogged samples. Asterisks (*) indicate a significant difference between well-watered and waterlogged samples at the same time point (*p* < 0.05).

The antioxidant enzyme activities in well-watered and waterlogged banana plants were measured at 1, 3, 5, 7, 14, and 24 days to determine the ROS scavenging mechanism of bananas towards waterlogging stress (Figure 4). Waterlogging stress significantly increased the superoxide dismutase (SOD) activity, producing the highest level of 0.112 U g^−1^ after one day of waterlogging (Figure 4A). However, the SOD activity decreased to its lowest level (0.0136 U g^−1^) after 24 days of waterlogging. The ascorbate peroxidase (APX) level of the waterlogged samples was significantly higher than well-watered samples after seven days of waterlogging (Figure 4B). The glutathione reductase (GR) enzyme activity for well-watered samples increased from 0.148 U g^−1^ on day 1 to 0.676 U g^−1^ on day 24. In contrast, a significant increase in the GR enzyme activity for waterlogged samples was observed on days 1, 5, and 7 (Figure 4C). The glutathione peroxidase (GPX) levels in waterlogged bananas were significantly higher than in well-watered plants on days 3, 14, and 24 of waterlogging, where the GPX level was highest on day 3 (0.287 µmol min^−1^ g^−1^) (Figure 4D). On the other hand, the catalase (CAT) activity was found to be like well-watered plants, with the highest recorded on day 7 for waterlogged bananas (0.120 nM cm^−1^ min^−1^ g^−1^) (Figure 4E).

### 2.5. Waterlogging Stress Altered Gene Transcription in Roots

**Figure 5:**
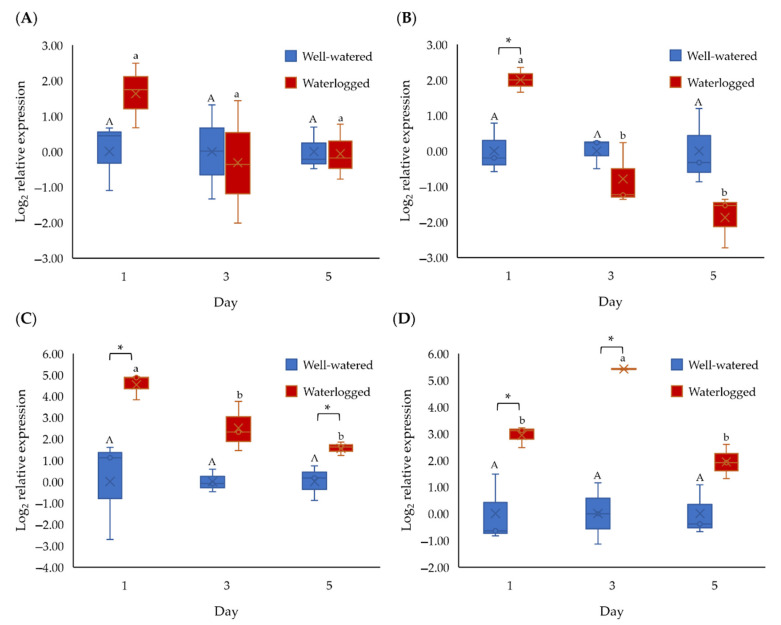
Gene expression of (**A**) *MaERFVII-1*, (**B**) *MaERFVII-2*, (**C**) *MaERFVII-3*, and (**D**) *ADH1* of banana for 1, 3, and 5 days of waterlogging. Error bars indicate standard error between biological replicates. Different capital letters indicate a significant difference between time points within well-watered samples. Different lowercase letters indicate a significant difference between time points within waterlogged samples. Asterisks (*) indicate a significant difference between well-watered and waterlogged samples at the same time point (*p* < 0.05).

We selected three *ERFVII*, namely *MaERFVII-1* (Macma4_09_g14450), *MaERFVII-2* (Macma4_08_g23860), and *MaERFVII-3* (Macma4_02_g02290), and *ADH1* (Macma4_02_g11450) genes for qPCR analysis to determine the early response of bananas toward waterlogging stress. These genes were selected as they are waterlogging-responsive genes and play a critical role in flooding tolerance. When banana plants were exposed to different waterlogging durations, we found that two out of three *ERFVII* genes were significantly (*p* < 0.05) upregulated on day 1 (Figure 5). The *ADH1* expression in waterlogged bananas was upregulated by 2.93- and 5.4- fold on day 1 and day 3, respectively, compared to well-watered bananas. We selected a 1-day waterlogging treatment for the subsequent RNA sequencing. The RNA samples with a RIN value of more than seven were sequenced (Table S2).

**Figure 6:**
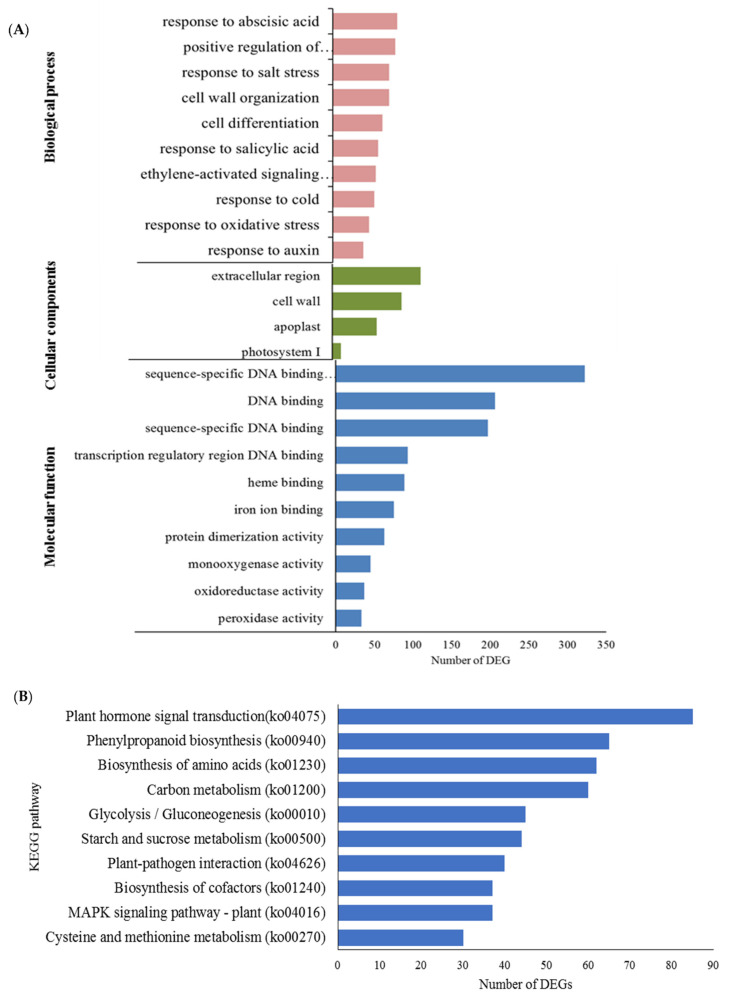
Functional categorization and enrichment of differentially changed genes between well-watered and waterlogged banana root samples. (**A**) Gene ontology enrichment according to biological processes, molecular functions, and cellular components and (**B**) the top ten significantly enriched KEGG pathways based on functional category.

The gene expression profiles of well-watered and waterlogged samples were analyzed using RNA-sequencing. Of the 407 million raw reads generated from the six cDNA libraries, approximately 406 million clean reads were obtained, ranging from 66.2 to 70.7 million reads per library (Table S3). The clean reads were then mapped to the banana genome retrieved from the banana genome hub DH Pahang (version 4.3) using Hisat2 (v2.0.1). The mapping rates of each library ranged from 80.37% to 83.54% (Table S4). Of the 3508 differentially expressed genes (DEGs), 1470 were upregulated, and 2038 were downregulated (Table S5). The identified unigenes were classified according to their gene ontology (GO) (Figure 6A). Most DEGs in the biological process category were involved in response to abscisic acid and salt stress and positive regulation of transcription. The cellular component category contained terms such as ‘nucleus’, ‘extracellular region’, and ‘cell wall’. The most dominant terms in the molecular function category were ‘sequence-specific DNA binding transcription factor activity’, ‘transcription regulatory region DNA binding’, and ‘heme binding’. Interestingly, both GO terms ‘positive regulation of transcription, DNA-templated’ and ‘cell wall organization’ had the highest number of upregulated DEGs, whereas ‘response to abscisic acid’ and ‘cell differentiation’ had the highest number of downregulated DEGs (Table 1).

There were 795 unigenes annotated according to the Kyoto Encyclopedia of Genes and Genome (KEGG) database [26], with 125 pathways significantly enriched. These include plant hormone signal transduction (ko04075), phenylpropanoid biosynthesis (ko00940), amino acid biosynthesis (ko01230), carbon metabolism (ko01200), and glycolysis (ko00010) (Figure 6B).

### 2.6. Hormone Signaling Pathways Are Differentially Regulated in Waterlogged Bananas

**Figure 7:**
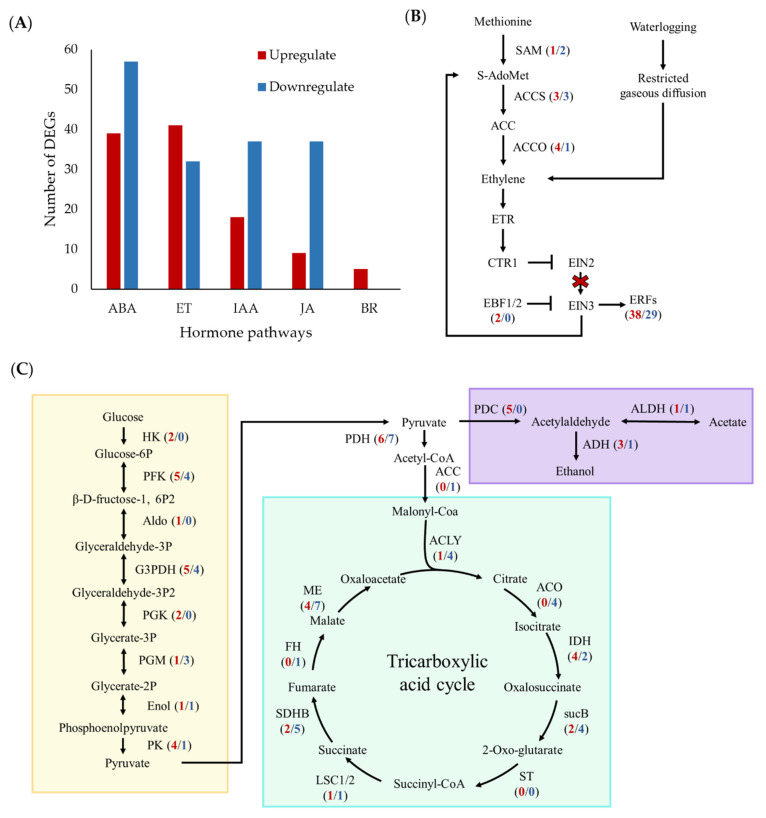
Plant hormone signal transduction pathway after 1 day of waterlogging stress. (**A**) The number of hormone-signal-related DEGs changes in bananas under waterlogging stress. The DEGs were involved in (**B**) the ethylene biosynthetic pathway and (**C**) carbon metabolism. The yellow box highlights the glycolysis pathway, leading to the ethanol fermentation in the purple box, and lastly, the blue box contains genes involved in the tricarboxylic acid (TCA) cycle. The arrows indicate activation of the gene, while the blunt end represents inhibition of the gene. The red cross indicates the inactivity of the EIN3 pathway that leads to the activation of ERF TFs. The numbers in parentheses correspond to the number of upregulated (red) and downregulated genes (blue). [ABA, abscisic acid; ACCO, 1-aminocyclopropane-1-carboxylic acid oxidase; ACCS, 1-aminocyclopropane-1-carboxylic acid synthase; ACLY, ATP citrate (pro-S)-lyase; ACO, aconitate hydratase; ADH, alcohol dehydrogenase; ALDH, aldehyde dehydrogenase; Aldo, aldolase; BR, brassinosteroid; CTR1, constitutive triple response 1; EBF, ethylene binding factor; ET, ethylene; EIN2/3, ethylene insensitive 2/3; Enol, enolase; IAA, auxin; FH, fumarate dehydrogenase; G3PDH, glyceraldehyde-3-phosphate dehydrogenase; HK, hexokinase; IDH, isocitrate dehydrogenase; JA, jasmonic acid; LSC1/2, succinate-CoA ligase; ME, malate dehydrogenase; PDC, pyruvate decarboxylase; PDH, pyruvate dehydrogenase; PEPC, phosphoenolpyruvate carboxykinase; PFK, phosphofructokinase; PGK, phosphoglycerate kinase; PGM, phosphoglycerate mutase; PK, pyruvate kinase; SAM, S-adenosyl-L-methionine; SDHB, succinate dehydrogenase; ST, succinyl transferase; sucB, 2-oxoglutarate dehydrogenase].

A total of 275 DEGs involved in plant hormone signaling transduction pathways were altered in waterlogged plants (Figure 7A). Specifically, 34.9% were associated with ABA signaling, 24.6% with ethylene signaling, 18.5% with auxin signaling, 15.5% with jasmonic acid, and 1.7% with brassinosteroid (Figure 7A). Genes enriched in the ABA pathway include bZIP domain-containing protein, abscisic acid-insensitive 5-like protein, abscisic receptor PYL, and protein phosphatase 2C. Of the 96 genes involved in this pathway, 39 were upregulated, and 57 were downregulated (Table S5). In the ethylene signaling pathway, upregulated genes include ethylene biosynthetic genes (*ACCO* and *ACCS*), ethylene transport receptors, and ethylene-responsive transcription factors (TFs) (Figure 7B). Enrichment analysis revealed that auxin-responsive genes, such as *SAUR32* (Macma4_02_g06820, Macma4_07_g20980), *IAA30*, and *IAA4*, were down-regulated. However, the auxin biosynthesis gene, namely *IAA-amino synthetase GH3.8* (Macma4_02_g16920 and Macma4_05_g01480), was upregulated by 2.4- and 3.4- log_2_ fold change. These results indicated that waterlogging could lead to a different hormone signaling regulation, which might play an important role in the waterlogging response and adaptation in bananas.

### 2.7. Hypoxia-Related Genes Showed Transcriptional Responses in Waterlogged Bananas

Pathways related to hypoxia and carbon metabolism were assessed to understand the changes in the waterlogging response in banana plants. All eight genes related to oxygen sensing, the plant cysteine oxidase (PCO) gene, were significantly upregulated by 1.45- to 5.0- fold in waterlogged samples (Table S5).

For carbohydrate metabolism, most genes related to glycolysis, including hexokinase, phosphofructokinase, aldolase, enolase, and glyceraldehyde-3-phosphate dehydrogenase, were upregulated (Figure 7C). The genes related to ethanol fermentation were upregulated in response to waterlogging, i.e., five pyruvate decarboxylase (PDC) genes and three alcohol dehydrogenase (ADH) genes. In contrast, most TCA cycle genes, namely citric synthase, aconitate hydratase, 2-oxoglutarate dehydrogenase, succinate dehydrogenase, fumarate dehydrogenase, and malate dehydrogenase, were down-regulated. These results indicate that fermentation is critical for bananas to respond to early waterlogging stress.

### 2.8. Expression of Transcription Factors (TF) Affected in Waterlogged Bananas

**Figure 8:**
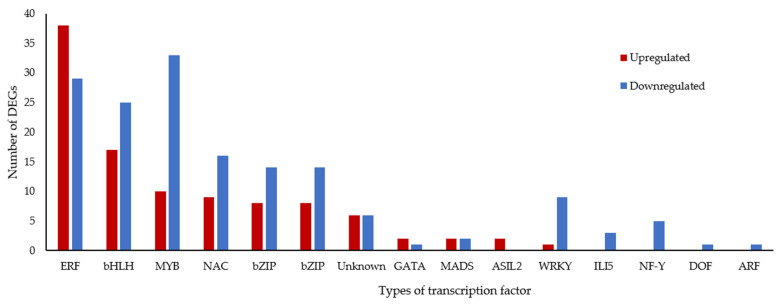
The number of waterlogging-related transcription factors that are classified according to their protein families. [ARF, auxin response factor; bHLH, basic helix-loop-helix; bZIP, basic zinc leucine zipper domain; DREB, dehydration-responsive element binding; ERF, ethylene response factor; MYB, myeloblastosis; NAC-NAM, ATAF, and CUC; ZF-TF, zinc finger transcription factor].

Among 3508 DEGs, a total of 267 were TFs, with 113 TFs being upregulated and 154 TFs being downregulated (*p* adjust < 0.01) (Figure 8). These TF families include AP2/ERF, bHLH, MYB, NAC, bZIP, and WRKY. Among ERF TFs, 38 were upregulated, whereas 29 TFs were downregulated (Figure 8). ERFVII gene (Macma4_11_g05400) was the most upregulated gene with a log_2_ fold change of 4.4, indicating that this TF might be critical in waterlogging responses (Table S5).

### 2.9. qPCR Validation of DEGs from the RNA-Sequencing

**Figure 9:**
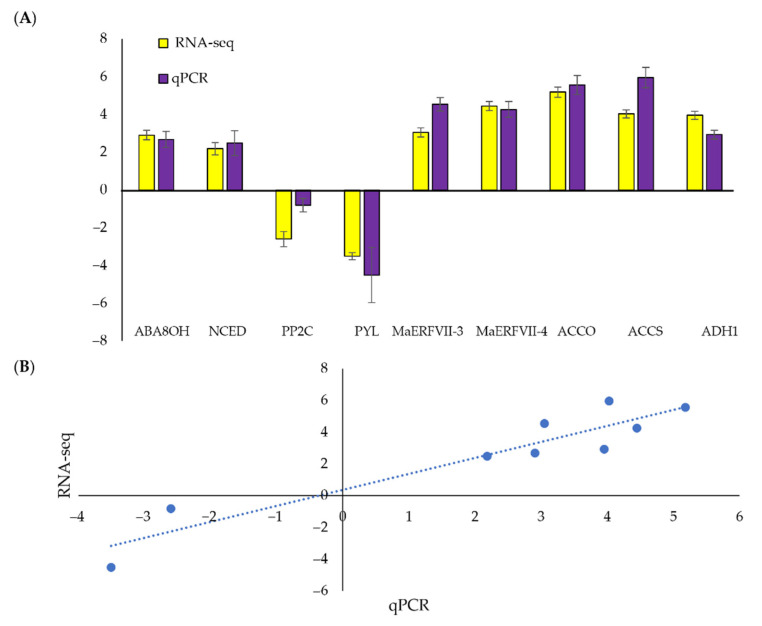
Comparison of RNA-sequencing and qPCR analyses. (**A**) The qPCR validation of up-and downregulated differentially expressed genes in waterlogged bananas relative to the well-watered bananas. Values are means ± SE of three biological replicates in qPCR. The expression levels of each gene are expressed relative to the mean values of the control samples. (**B**) The correlation between qPCR and RNA-sequencing data. [ABA8OH, abscisic acid 8′ hydroxylase; ACCO, 1-aminocyclopropane-1-carboxylic acid oxidase; ACCS, 1-aminocyclopropane-1-carboxylic acid synthase; ADH1, alcohol dehydrogenase 1; NCED, 9-cis-epoxycarotenoid dioxygenase; PYL, PYR-like proteins; PP2C, type 2C protein phosphatase].

Nine genes involved in the abscisic acid (ABA) biosynthetic pathway, ethylene signaling pathway, and a hypoxia-responsive gene were selected for qPCR analysis to validate the transcriptomic data (Figure 9A). The expression of the selected genes between RNA-sequencing and qPCR were highly correlated, with a correlation coefficient of 0.9408 (Figure 9B).

## 3. Discussion

### 3.1. Morphological Responses of Bananas Subjected to Waterlogging

As sessile organisms, plants are unable to escape when challenged by unfavorable environmental conditions. However, they can deploy complex morpho-physiological and molecular mechanisms to cope and adapt to these adverse environmental stresses [11]. In this study, the banana plants elicited morphological responses to combat the imposed waterlogging stress to increase their chances of survival. Waterlogged soils progressively exert a detrimental effect on plant root respiration and nutrient uptake. This phenomenon resulted in leaf wilting, which was observed at the latter stages of waterlogging. However, we did not observe significant changes in leaf area and RWC. One of the possible explanations is that the experimental periods might be too short to evaluate the changes in leaf area. In contrast, no significant changes in RWC might be because of the driving force generated by banana plants for water charging of cellular water storage capacities [27]. Although the mechanism is unclear, this process might improve the water uptake by plants and the water status of leaves. On the other hand, we found that waterlogging induced adventitious roots and aerenchyma formation. These results concurred with previous studies, which showed that waterlogging significantly increases the number of adventitious roots and aerenchyma formation [28,29]. The formation of aerenchyma is essential in plant waterlogging tolerance as it can provide oxygen to the submerged organs of a plant [30]. On the other hand, adventitious root formation might be the result of ethylene entrapment due to waterlogged soils, causing auxin accumulation in the stem [31].

### 3.2. Oxidative Stress and Antioxidant Changes in Roots of Waterlogged Bananas

Waterlogging stress generally leads to the accumulation of MDA and H_2_O_2_. Our findings agree with previous studies, where MDA and H_2_O_2_ were elevated under waterlogging or hypoxia stress in various plants [32,33]. MDA is the end product of lipid peroxidation, frequently used to determine ROS-induced oxidative injury [34]. The significant differences in MDA levels at various treatment times indicate that MDA levels in bananas are time-specific. The waterlogged bananas generally produced higher MDA than their well-watered counterparts over time, despite having increased antioxidant activities. One of the possible explanations for this is that the antioxidant enzymes might provide a short-term defense response against stress, and prolonged waterlogging stress might cause mitochondria dysfunction and cell membrane damage, as observed in the MDA assay [35]. In contrast, another ROS scavenger, proline, showed no significant changes between treatments and time points. Its production is widely observed when plants are exposed to waterlogging stress to maintain protein structural stability [36], as shown in various plants, such as peach and cucumber [37,38].

The excessive accumulation of ROS accompanied by increasing antioxidant enzyme activities. In the present study, the increased antioxidant enzyme activities in the banana roots under waterlogging indicated that they possess efficient ROS scavenging systems. For example, the SOD activity remained high since day 1 of waterlogging, reaffirming the role of SOD as the first line of defense against ROS. In plants, APX and CAT are predominant enzymes in alleviating the damage caused by stresses [32]. This study recorded a remarkable increase in APX activity in waterlogged banana roots. Similar findings were also reported by Xie et al. [39] and Da-Silva et al. [40], where the waterlogging stress increased APX activity. In contrast, the CAT activity for well-watered and waterlogged samples was not significantly different. We speculate that no significant difference in CAT activity is partly the result of the increased APX activity, as APX has a higher affinity for H_2_O_2_ than CAT [41]. In addition, the increasing trend in H_2_O_2_ accumulation consequent to increased stress duration might also contribute to this observation [42].

GPX is an oxygen radical scavenger, catalyzing the reduction of H_2_O_2_ via glutathione to generate water and glutathione disulphide. Consequently, GR reduces glutathione disulphide to glutathione, supplying electron donors for the subsequent detoxification of H_2_O_2_ [43]. Our results showed that GPX and GR activities in waterlogged banana roots were generally higher than in well-watered roots, which is supported by other studies [42,44]. Taken together, the enhanced antioxidant enzyme system, especially SOD, helps banana plants cope with accumulated ROS under waterlogging conditions.

### 3.3. Oxygen Sensing of Banana under Waterlogging Stress

Waterlogging stress affects oxygen-sensing in plants. We found that the expression of PCOs and ERFVIIs, which represent the oxygen-sensing machinery for plant survival under flooding, was significantly altered. Under normal conditions, ERFVII TF N-terminal cysteine residue is subjected to O_2_-dependent degradation through the N-end rule pathway by PCO [45]. However, in the absence of oxygen, PCO cannot destabilize ERFVII TFs, leading to the activation of hypoxia-responsive genes [46]. Surprisingly, six PCO genes in banana roots were upregulated under waterlogging stress. We speculate that this might be due to the residual oxygen molecules present in the plants or water. Loreti et al. [47] showed that the *PCO2* expression in *A. thaliana* was upregulated under anoxia stress but reduced to a minimum level after 24 h of treatment.

Ethylene response factor (ERFs) are a critical flooding-responsive mechanism in plants. They are constitutively expressed but are degraded in the presence of oxygen [48]. Our results showed that among 67 ERF TFs, those in the IIa, IIIb, Va, VII, VIIIa, IX, and X subgroups were upregulated, while ERF TFs in IIb, Vb IXc, and Xa subgroups were downregulated. In addition, both IIb and IIId subgroups contained upregulated and downregulated transcripts. It is noteworthy that six out of 18 genes of the ERFVII family were upregulated. Members of the ERFVII are well-known as the main regulator of plant responses to low oxygen conditions [49]. For instance, the knockout of *erfVII* in *A. thaliana* decreased its tolerance to hypoxia [50]. In wheat, the overexpression of ERFVII improved the waterlogging tolerance without grain yield penalty [51].

It has been well documented that ethylene accumulation is a key signal for flooding. Our transcriptome data show that several ethylene biosynthesis genes, such as S-adenosyl-L-methionine (SAM) and 1-aminocyclopropane-1-carboxylic acid oxidase (ACCO), were upregulated in the submerged roots. These results agree with previous studies showing that waterlogging stress promotes ethylene biosynthesis [52]. However, although ethylene biosynthesis genes were upregulated, waterlogging stress did not affect the downstream genes in the ethylene pathway, such as *ETR*, *CTR1*, and *EIN3*. Furthermore, although the increased EBF transcription could lead to EIN3 inhibition, this inhibition did not decrease the transcription of ERF TFs. Hence, we speculate that an EIN3-independent pathway might be involved in activating ERF TF transcription since there is growing evidence of the EIN3/EIL1-independent mediated pathway in the ethylene pathway. For example, the double knockout *ein3ein1* Arabidopsis mutants were able to respond to ethylene under stress [53]. However, further studies are required to verify this.

### 3.4. Expression in Genes Involved in ABA Pathways of Bananas under Waterlogging Stress

Several genes associated with the ABA pathway were found to have a significant transcriptional response to the waterlogging stress. For instance, the gene encoding the rate-limiting enzyme in ABA biosynthesis, 9-cis-epoxycarotenoid dioxygenase 1 (NCED1), was downregulated, while the ABA degradation enzyme, ABA 8′-hydroxylase gene, was upregulated. ABA is a phytohormone known to respond to drought, salinity, and cold stress, and the reduced ABA content is a common response to waterlogging. A decrease in ABA would trigger ethylene accumulation and subsequently stimulate adventitious root and aerenchyma development in submerged plants [54,55]. The exogenous ABA treatment has been demonstrated to inhibit adventitious root primordia formation in tomatoes [54] and aerenchyma formation in soybean roots [56]. Besides, a decrease in ABA is essential for waterlogged-induced gibberellic acid to promote shoot elongation [45]. These results indicate that ABA acts as a negative regulator in waterlogging tolerance in plants.

### 3.5. Carbon Metabolism Is Essential for Energy Supply during Waterlogging

When plants are exposed to hypoxic conditions, they immediately alter their gene transcription to synthesize anaerobic polypeptides, deriving from glycolysis and ethanol fermentation [57]. This study found that glycolysis-related genes were significantly expressed on one day of waterlogging. These include hexokinase, phosphoglycerate kinase, and pyruvate kinase. Given the need to maintain sufficient energy for plant survival since nutrient uptake is inhibited in hypoxic roots, it is not surprising that carbon metabolism was differentially altered under waterlogging stress. Similarly, increased transcription of genes related to starch metabolism and the glycolysis/gluconeogenesis pathways has been reported in the waterlogged roots of *Cerasus sachalinensis* [58] and barley [59].

Aerobic respiration in roots is limited under low oxygen conditions, resulting in glycolysis being channeled to fermentative pathways. These changes are necessary to produce energy for cell survival. Indeed, PDC and ADH were upregulated in waterlogged bananas. Pyruvate generated from glycolysis is metabolized to ethanol via PDC and ADH. This process releases energy in the form of adenosine triphosphate (ATP) to increase the chances of plant survival in the absence of aerobic respiration.

Genes associated with the TCA, such as glutamate synthase (GS), glutamate dehydrogenase (GDH), and glutamate carboxylase, were downregulated in the roots of waterlogged banana plants. This finding concurred with the study by Ren et al. [60], where the authors showed that the activity of GS and GDH was reduced in the stems and leaves of waterlogged maize. The changes in carbon metabolism are often accompanied by changes in nitrogen metabolism to maintain the carbon-nitrogen balance. The increase of amino acid catabolism is required to compensate for the insufficient energy supply during stress. This can be seen in the waterlogged banana roots, where the alanine biosynthesis gene (alanine aminotransferase; AlaAT) was upregulated. Alanine is a carbon and nitrogen source, and its biosynthesis is crucial for plants to survive waterlogging [61]. These results suggest that activation of amino acid catabolism and alcohol fermentation are vital to fulfilling the energy demand in plants under waterlogging stress.

### 3.6. A Model for the Response of Banana Plants to Waterlogging

**Figure 10:**
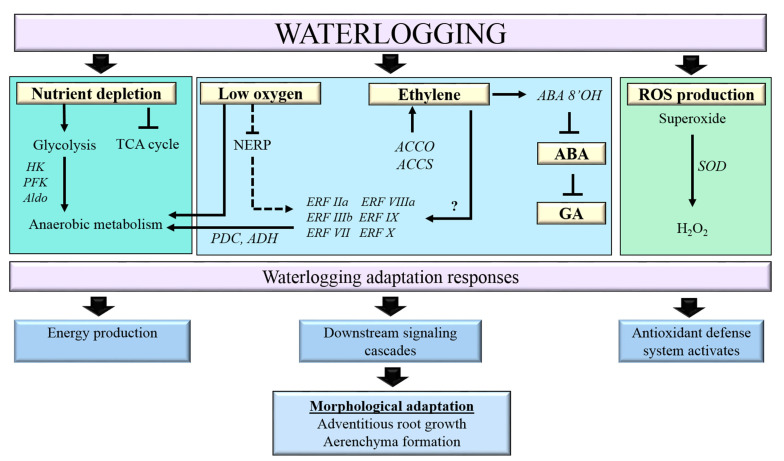
The proposed model of the pathways involved in *Musa acuminata* under waterlogging stress. Arrows indicate the activation of the pathway, while blunt end arrows indicate inhibition of the pathway. The dashed line indicates partial activation of the NERP pathway, leading to the partial inhibition of ERFVII TFs. The question mark indicates an unknown EIN3/EIL independent pathway that activates ERF genes. [ABA, abscisic acid; ABA 8′OH, abscisic acid 8′ hydroxylase; ACCO, 1-aminocyclopropane-1-carboxylic acid oxidase; ACCS, 1-aminocyclopropane-1-carboxylic acid synthase; Aldo, aldolase; ERF, ethylene response factor; GA, gibberellic acid; H_2_O_2_, hydrogen peroxide; HK, hexokinase; NERP, N-end rule pathway; PDC, pyruvate decarboxylase; PFK, phosphofructokinase; SOD, superoxide dismutase; TCA, tricarboxylic acid].

We propose a model for waterlogging responses in banana plants (Figure 10). Waterlogging affects changes in plant cells, such as limitation of gas diffusion, nutrient depletion, and increased ROS production. To respond and adapt to waterlogging, banana plants have evolved to rapidly elongate their adventitious roots and aerenchyma formation and activate the antioxidant defense system. Our data revealed transcriptional changes in the downstream components of hormone signaling and carbon and energy metabolisms during waterlogging.

## 4. Materials and Methods

### 4.1. Plant Material and Waterlogging Treatment

Two-month-old banana (*M. acuminata* cv. Berangan) seedlings purchased from the MB Tissue Culture Resources, Shah Alam, Selangor, Malaysia, were acclimatized in a greenhouse at the Universiti Malaya. A total of 108 banana plantlets grown in flowerpots containing soil with equal portions of coco peat, sand, organic matter, and black soil were transferred to a 3 L plastic container with 18 cm diameter. The plants were maintained under natural light (12 h photoperiod) at a controlled temperature (25 ± 2 °C) under a relative humidity of 65 ± 5%. Waterlogging treatment was conducted by filling the plastic containers with water 2 cm above the soil surface. The containers were refilled with tap water to maintain a constant water level. The well-watered bananas were planted in well-drained soil and watered once a day during treatment. The waterlogging treatments were conducted for 1, 3, 5, 7, 14, and 24 days. Each time point consisted of nine plants (*n* = 9), with three plants in a plastic container. After treatment, plants were carefully dug and washed with tap water to remove dirt attached to the roots. The morphological changes of the treated and non-treated banana plants were determined for each time point. After waterlogging treatment, root samples were harvested, ground into a fine powder in the presence of liquid nitrogen and stored at −80 °C until use. These samples were used for the subsequent gene expression analysis and biochemical assays.

### 4.2. Morphological Analysis

Morphological changes, such as total root length, R/S DW ratio, leaf area, and leaf relative water content (RWC), were measured. The total root length was determined based on the sum of the length of each root. Each root was measured using a meter ruler. The plants were divided into the shoot and root parts and oven-dried at 80 °C for two weeks or until constant weight for dry mass. The R/S DW ratio was calculated based on the dry matter of the root and shoot parts of the plants.

The leaf area differences were calculated based on the differences between leaf area measurements before and after the waterlogging treatment. The RWC was measured according to Turner [62]. For RWC, the first mature leaf was excised into 2 cm × 2 cm size before measuring the total fresh weight. Next, leaves were submerged in distilled water, and turgid weight was measured after 24 h. For DW, leaves were dried in a 60 °C oven for 24 h or until a constant weight was achieved. RWC was calculated according to the following equation:

RWC (%) = (FW − DW)/(TW − DW) × 100%(1)

FW = Fresh weight (g)
DW = Dry weight (g)
TW = Turgid weight (g)

### 4.3. Adventitious Root and Aerenchyma Formation

**Figure 11:**
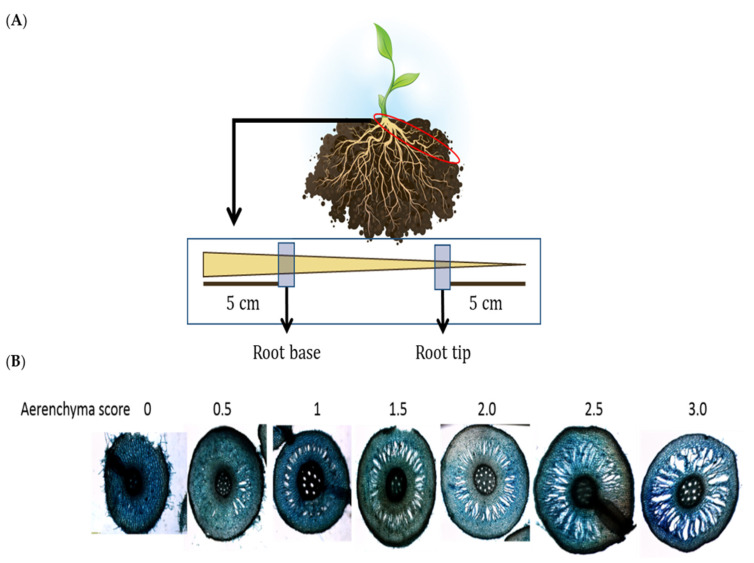
Determination of root aerenchyma formation. (**A**) The roots were removed from the plant and excised 5 cm from the root base, which is closer to the stem, and 5 cm at the root tip, which is away from the plant. (**B**) The aerenchyma score was assessed according to the descriptions from Mano and Omori [63].

Adventitious roots were calculated based on the number of roots per plant protruding from the banana stem. Aerenchyma formation was assessed according to Mano et al. [63]. A total of three lateral roots with a root length of more than 15 cm were randomly selected from each banana plant in each treatment group to determine the presence of aerenchyma. Cross-sections of roots about 1 cm thick were made using a handheld razor blade 5 cm beyond the root base and 5 cm beyond the root tip (Figure 11A). Aerenchyma in the root cortex was visually scored under an inverted fluorescent microscope (Olympus Co., Tokyo, Japan) on a scale of 0 to 3, where 0 indicates no aerenchyma, 1 indicates radial aerenchyma formation, 2 indicates radial formation extended to the epidermis, and 3 indicates well-formed aerenchyma (Figure 11B).

### 4.4. Malondialdehyde, Proline, and Hydrogen Peroxide

H_2_O_2_ content was measured as described by Junglee et al. [64]. Frozen banana root tissues (150 mg) were homogenized with 1 mL of buffer solution containing 0.025% (*w*/*v*) TCA, 0.5 M potassium iodide, and 2.5 mM potassium phosphate buffer. The absorbance of the supernatant was measured at 350 nm using a spectrophotometer. The H_2_O_2_ content was determined using the standard curve prepared from the serial dilution of H_2_O_2_. Malondialdehyde (MDA) content was determined as described by Amnan et al. [34]. The root tissues were homogenized and incubated with 0.5% (*w*/*v*) thiobarbituric acid (TBA) in 20% (*w*/*v*) trichloroacetic acid (TCA) at 95 °C for 15 min. The solution was then placed on ice to terminate the reaction and centrifuged to collect the supernatant. A spectrophotometer was used to measure the absorbance of the solution at 532 and 600 nm, where the net absorbance value was recorded. Lastly, the MDA content was calculated with the extinction-coefficient 155 mM^−1^ cm^−1^.

Proline content was estimated as described by Bates et al. [65]. A total of 0.5 g root samples were homogenized in 1 mL of 70% ethanol. The filtrate was mixed with an equal volume of acid-ninhydrin and acetic acid for 1 h at 100 °C. The reaction was terminated using an ice bath. The solution was partitioned against 4 mL toluene and measured in the organic layer for absorbance at 520 nm. Commercially available proline was used as a standard to construct a calibration curve.

### 4.5. Antioxidant Enzyme Assays

The antioxidant enzyme activities, namely SOD, APX, GR, GPX, and CAT, in well-watered and waterlogged banana plants, were measured. The antioxidant enzyme activities were determined according to Zhang et al. [66]. In brief, soluble proteins were extracted from the homogenized roots in potassium phosphate buffer (pH 7.0) containing ethylenediaminetetraacetic acid (EDTA) and polyvinylpyrrolidone, with the addition of ascorbic acid in the case of APX assay. The supernatant was collected through centrifugation and used for SOD, APX, CAT, GR, and GPX assays.

Total SOD activity was monitored through the inhibition of photochemical reduction of nitro blue tetrazolium. The reaction mixture was illuminated for 10 min under a 35 W fluorescent tube. The reaction was then terminated by placing the tube in a dark container. The absorbance of the mixture at 560 nm was measured for all samples at 15 s intervals for 3 min. The SOD level was calculated according to the following formula:

SOD (U g^−1^) = ∆A/Blank A_560nm_ × (Volume of reaction)/(Volume of enzyme) × 0.02 × 1/FW × 100 (2)

∆A = Difference in absorbance between blank and sample
FW = Sample fresh weight (g)

APX activity was determined from the decrease in absorbance at 290 nm reading for 1 min in a reaction mixture with an extinction coefficient of 28 mM^−1^ cm^−1^. The APX reaction mixture contained enzyme extract, 50 mM phosphate buffer (pH 7.0), 0.5 mM ascorbic acid, and 0.1 mM H_2_O_2_. GR assay was performed according to Carlberg et al. [67] through the oxidation of 1 µmol of NADPH in 3 min at 290 nm with an extinction coefficient of 6200 M^−1^ cm^−1^. The GR reaction mixture contained enzyme extract, 50 mM phosphate buffer (pH 7.0), 0.5 mM EDTA, 0.1 mM reduced nicotinamide adenine dinucleotide phosphate (NADPH), and 1 mM glutathione disulphide (GSSG). GPX was measured as the oxidation of guaiacol in the presence of H_2_O_2_ with an extinction coefficient of 43.6 M^−1^ cm^−1^. The reaction mixture consisted of enzyme extract, 100 mM phosphate buffer (pH 7.0), guaiacol, and 0.1 mM H_2_O_2_. The reaction started after adding guaiacol, followed by changes of 470 nm over 3 min [68]. CAT activity was monitored through the consumption of H_2_O_2_ with an extinct coefficient of 39.4 mM^−1^ cm^−1^, resulting in a decrease in absorbance at 240 nm for 3 min. The CAT reaction mixture consisted of enzyme extract, 50 mM phosphate buffer (pH 7.0), and 10 mM H_2_O_2_. The enzyme activities were calculated according to the general formula:

Enzyme activity (M min^−1^ g^−1^ FW) = (∆A × V_TR_)/(ϵ × ∆t × 1 cm × V_e_ × g FW) × 1000 (3)

∆A = Difference in absorbance of mixture
V_TR_ = Volume of reaction (mL)
V_e_ = Volume of enzyme extract (mL)
∆t = Difference in time of absorbance (min)
ϵ = extinction coefficient
FW = Sample fresh weight (g)

### 4.6. RNA Extraction

Total RNA was extracted from the roots using the CTAB extraction method, according to Asif et al. [69]. The RNA quality was analyzed using Nanophotometer Perl^®^ (Implen GmbH, Munich, Germany), whereas the RNA integrity was visualized using agarose gel electrophoresis and Bioanalyzer. The extracted RNA was treated with DNase (Qiagen, Hilden, Germany) according to the manufacturer’s protocol to remove residual genomic DNA.

### 4.7. Quantitative Real-Time PCR

Quantitative real-time PCR (qPCR) was performed to analyze the expression of ERFVII TFs. The first strand of cDNA was synthesized from total RNA (2 µg) using the NxGen M-MuLV Reverse Transcriptase cDNA synthesis kit (LGC Biosearch Technologies, Hoddesdon, UK). The qPCR consisted of a final volume of 15 μL containing 10 ng of cDNA, 0.3 µM primers, and 2 × SG Fast qPCR Master Mix (Sangon Biotech Co., Ltd. Shanghai, China), with the elongation factor 1-alpha and tubulin as reference genes (Table S6). The qPCR was carried out according to the manufacturer’s protocol. Relative expression levels were calculated according to Pfaffl [70]. The qPCR analysis was carried out with three biological replicates and three technical replicates for each gene.

### 4.8. RNA Sequencing

The extracted RNA with a RIN value between 6.5 and 10 was used for poly-A enriched library construction. RNA sequencing was performed on an Illumina system with paired-end 150 bp reads. Raw reads were assessed for their quality using the FastQC program (version 0.11.3) (Cambridge, UK) [71] and filtered using Cutadapt (v1.9.1) (Uppsala, Sweden) [72]. Cutadapt software was used to remove primer and adapter sequences from the raw reads to obtain high-quality clean data. The clean reads of all samples were aligned against the banana reference genome (*M. acuminata* DH-Pahang version 4.3) (https://banana-genome-hub.southgreen.fr/content/download, (accessed on 25 November 2021) [73]. The mapping was performed using Hisat2 (v2.0.1) [74] using the default parameters. DEGs were identified using the DESeq2 Bioconductor package [75]. GOSeq (v1.34.1) [76] was used to identify GO terms that annotate a list of enriched genes, while the KEGG [26] was used to enrich genes in the KEGG pathway. The generated FASTQ files were deposited at the NCBI Sequence Read Archives database (https://www.ncbi.nlm.nih.gov/sra, (accessed on 25 July 2022) under BioProject ID: PRJN850880.

### 4.9. Statistical Analysis

The morphological experiments were conducted with three replicates per treatment and were repeated thrice. The biochemical and RNA sequencing experiments consisted of three biological replicates with three technical replicates for each biological replicate. Data are presented as means ± standard error. All morphological and biochemical data were recorded and analysed using one-way ANOVA with Duncan’s multiple range comparison post-hoc test at a significance level of *p* < 0.05 using SPSS 23.0 (SPSS, Chicago, IL, USA).

## 5. Conclusions

This study describes the morphological and molecular responses of banana plants in response to waterlogging stress. In general, the waterlogging-stressed plants showed a higher number of adventitious roots, enlarged root aerenchyma, and enhanced antioxidant enzyme activities than well-watered plants. The early waterlogging-responsive mechanisms of bananas were determined by examining their transcriptome changes. Several unigenes were validated to be differentially expressed, and most of them were classified as abiotic stress-related transcription factors, signal transduction, and carbohydrate metabolisms. Although the ethylene biosynthetic pathway was upregulated, the ethylene-independent pathway activating ERF TFs might also be involved. Our findings provide insight into the complex molecular events involved in response to waterlogging stress of banana roots, which could help develop waterlogging resilient crops for the future climate.

## Figures and Tables

**Table 1:** The GO terms of banana 24 h after waterlogging stress.

GO ID	GO Term	*p*-Value	False Discovery Rate	Upregulated DEG
GO:0045893	Positive regulation of transcription, DNA-templated	5.40 × 10^−8^	2.80 × 10^−5^	38
GO:0071555	Cell wall organization	3.37 × 10^−5^	6.16 × 10^−3^	38
GO:0009737	Response to abscisic acid	7.20 × 10^−9^	4.07 × 10^−6^	31
GO:0009873	Ethylene-activated signaling pathway	5.98 × 10^−8^	2.86 × 10^−5^	29
GO:0009651	Response to salt stress	4.82 × 10^−4^	4.83 × 10^−2^	26
GO:0006979	Response to oxidative stress	4.18 × 10^−6^	1.24 × 10^−3^	21
GO:0009409	Response to cold	2.61 × 10^−4^	3.12 × 10^−2^	17
GO:0010200	Response to chitin	4.45 × 10^−5^	7.28 × 10^−3^	16
GO:0010411	Xyloglucan metabolic process	9.77 × 10^−6^	2.43 × 10^−3^	16
GO:0042546	Cell wall biogenesis	4.29 × 10^−5^	7.21 × 10^−3^	16
GO:0009751	Response to salicylic acid	1.17 × 10^−13^	1.45 × 10^−10^	15
GO:0042744	Hydrogen peroxide catabolic process	1.22 × 10^−6^	3.98 × 10^−4^	14
GO:0030154	Cell differentiation	9.60 × 10^−7^	3.31 × 10^−4^	12
GO:0042545	Cell wall modification	2.81 × 10^−4^	3.29 × 10^−2^	12
GO:0009611	Response to wounding	2.00 × 10^−6^	6.22 × 10^−4^	11
GO:0055114	Oxidation-reduction process	4.77 × 10^−5^	7.61 × 10^−3^	11
GO:0009753	Response to jasmonic acid	6.59 × 10^−6^	1.86 × 10^−3^	9
GO:0009693	Ethylene biosynthetic process	2.65 × 10^−5^	5.14 × 10^−3^	9
GO:0009835	Fruit ripening	1.59 × 10^−4^	2.30 × 10^−2^	9
GO:0009741	Response to brassinosteroid	2.09 × 10^−4^	2.74 × 10^−2^	9
GO ID	GO Term	*p*-Value	False Discovery rate	Downregulated DEG
GO:0009737	Response to abscisic acid	7.20 × 10^−9^	4.07 × 10^−6^	51
GO:0030154	Cell differentiation	9.60 × 10^−7^	3.31 × 10^−4^	51
GO:0009651	Response to salt stress	4.82 × 10^−4^	4.83 × 10^−2^	46
GO:0009751	Response to salicylic acid	1.17 × 10^−13^	1.45 × 10^−10^	43
GO:0045893	Positive regulation of transcription, DNA-templated	5.40 × 10^−8^	2.80 × 10^−5^	42
GO:0009409	Response to cold	2.61 × 10^−4^	3.12 × 10^−2^	36
GO:0071555	Cell wall organization	3.37 × 10^−5^	6.16 × 10^−3^	34
GO:0009733	Response to auxin	3.92 × 10^−4^	4.06 × 10^−2^	31
GO:0009873	Ethylene-activated signaling pathway	5.98 × 10^−8^	2.86 × 10^−5^	26
GO:0006979	Response to oxidative stress	4.18 × 10^−6^	1.24 × 10^−3^	25
GO:0009753	Response to jasmonic acid	6.59 × 10^−6^	1.86 × 10^−3^	25
GO:0009611	Response to wounding	2.00 × 10^−6^	6.22 × 10^−4^	24
GO:0009813	Flavonoid biosynthetic process	1.47 × 10^−5^	3.20 × 10^−3^	20
GO:0055114	Oxidation-reduction process	4.77 × 10^−5^	7.61 × 10^−3^	18
GO:0042744	Hydrogen peroxide catabolic process	1.22 × 10^−6^	3.98 × 10^−4^	17
GO:0071365	Cellular response to auxin stimulus	2.58 × 10^−7^	1.07 × 10^−4^	15
GO:0031408	Oxylipin biosynthetic process	3.68 × 10^−5^	6.53 × 10^−3^	15
GO:0010200	Response to chitin	4.45 × 10^−5^	7.28 × 10^−3^	14
GO:1901332	Negative regulation of lateral root development	2.33 × 10^−9^	1.45 × 10^−6^	12
GO:0010345	Suberin biosynthetic process	1.34 × 10^−5^	3.20 × 10^−3^	11

## Data Availability

Sequencing data are available at the National Center for Biotechnology Information (NCBI) under BioProject ID: PRJNA850880.

## Supplementary Materials

The following supporting information can be downloaded at: https://www.mdpi.com/article/10.3390/plants11152052/s1, Figure S1: Representative image of banana plants exposed to waterlogging stress for 0, 1, 3, 5, 7, 14, and 24 days. A total of 108 banana plantlets grown in flowerpots were transferred to a 3 L plastic container with 18 cm diameter. Each time point consisted of nine plants (*n* = 9), with three plants in a plastic container. Waterlogging treatment was conducted by filling the plastic containers with water 2 cm above the soil surface. The containers were refilled with tap water to maintain a constant water level; Table S1: The shoot and root DW of banana plants in response to waterlogging stress; Table S2: The RIN value, 28S/18S ratio, and concentration of RNA prior to library preparation; Table S3: Summary of the raw and cleaned reads; Table S4: Total reads and mapped reads for well-watered and waterlogged bananas; Table S5: The list of significantly differentially expressed genes; Table S6: Sequences of primers, banana genome hub gene ID, and the product length of the targets.
