# Supplementary material for: Waterlogging Stress Induces Antioxidant Defense Responses, Aerenchyma Formation and Alters Metabolisms of Banana Plants
Source: Plants (Basel). 2022 Aug 5;11(15):2052. doi: 10.3390/plants11152052 (PMC9370344; doi:10.3390/plants11152052)
Supplement: Supplementary file 1 [file plants-11-02052-s001.zip › Figure S1.pptx]

## Slide 1
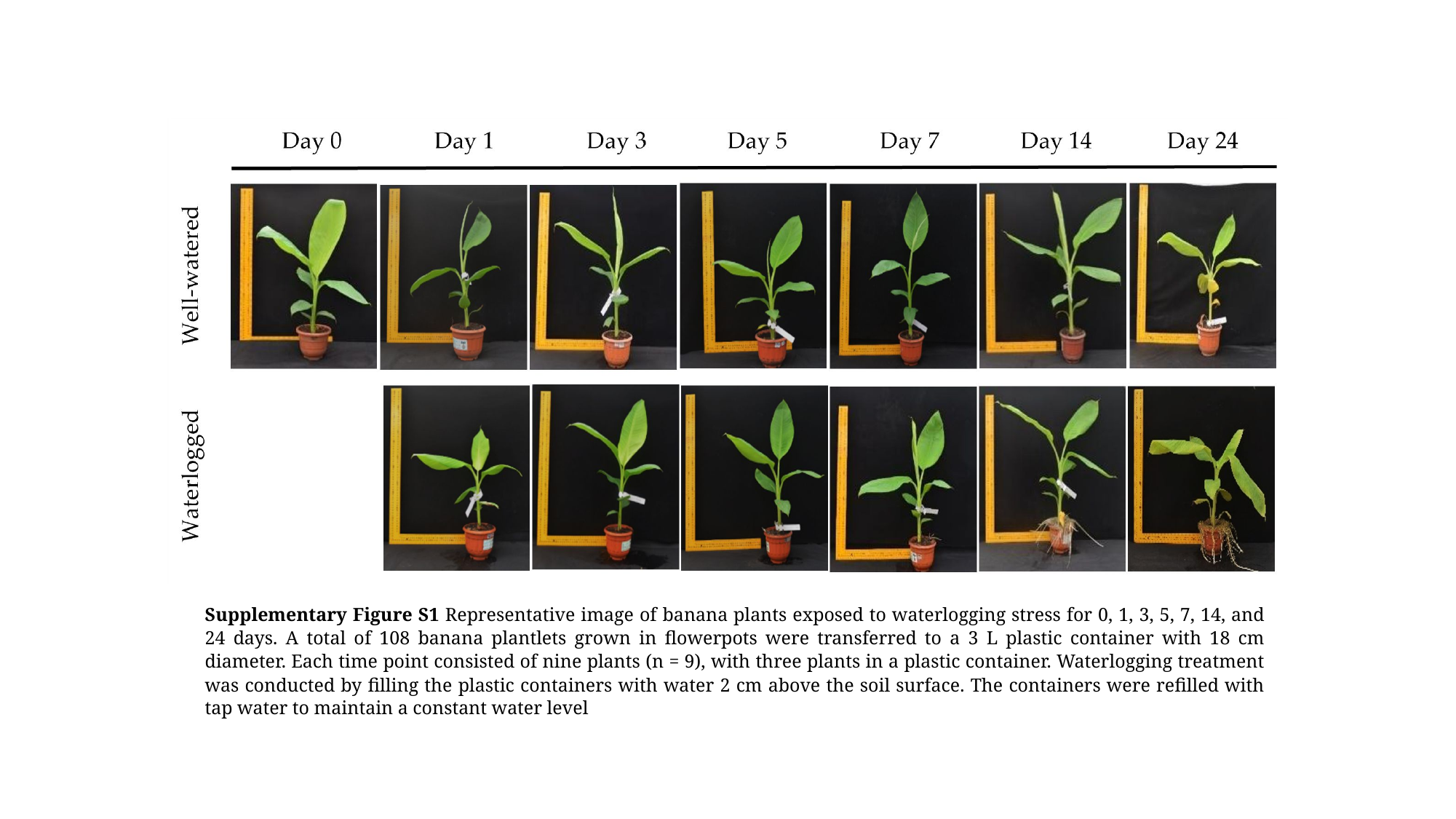

Supplementary Figure S1 Representative image of banana plants exposed to waterlogging stress for 0, 1, 3, 5, 7, 14, and 24 days. A total of 108 banana plantlets grown in flowerpots were transferred to a 3 L plastic container with 18 cm diameter. Each time point consisted of nine plants (n = 9), with three plants in a plastic container. Waterlogging treatment was conducted by filling the plastic containers with water 2 cm above the soil surface. The containers were refilled with tap water to maintain a constant water level
